# The Complete Chloroplast Genome of *Erythrina variegata* L. (Papilionoideae, Fabaceae)

**DOI:** 10.1002/ece3.70838

**Published:** 2025-01-10

**Authors:** Nguyen Nhat Nam, Nguyen Ngoc Trai, Nguyen Phuong Thuy, Le Quoc Duy, Pham Nguyen Tuong Van, Tan Tai Nguyen, Hoang Dang Khoa Do

**Affiliations:** ^1^ School of Agriculture and Aquaculture Tra Vinh University Tra Vinh City Vietnam; ^2^ College of Medicine and Pharmacy Tra Vinh University Tra Vinh City Vietnam; ^3^ Biotechnology Institute Tra Vinh University Tra Vinh City Vietnam; ^4^ Functional Genomics Research Center, NTT Hi‐Tech Institute Nguyen Tat Thanh University Ho Chi Minh City Vietnam

**Keywords:** comparative genomics, Indian coral tree, phylogeny, plastome

## Abstract

*Erythrina variegata* L. 1754, a thorny deciduous tree of Fabaceae, contains various chemical compounds such as alkaloids, flavonoids, and triterpenoids and exhibits anti‐depressant, anti‐inflammatory, and antidiabetic activities. However, genomic data of *E. variegata* are limited. In this study, the complete chloroplast genome of *E. variegata* was sequenced and characterized using Illumina sequencing platform. The chloroplast genome of *E. variegata* was 152,351 bp in length and consisted of a large single copy (82,907 bp), a small single copy (26,309 bp), and two inverted repeat regions (16,826 bp). There were 79 protein‐coding genes, 30 transfer RNA genes, and four ribosomal RNA genes. Comparative analysis revealed high conservation of chloroplast genomes among *Erythrina* species regarding genome size, structure, and gene content. The phylogenetic study also indicated a close relationship between *E. variagata* and *E. sanwicensis*. This study provides initial plastome data for further genomic studies examining *E. variegata* and related species in Fabaceae.

## Graphical abstract

This study reports the complete chloroplast genome of *Erythrina variegata* and its features. Also, comparative genomic analysis was conducted to explore the genomic diversity among Erythrina species. The phylogenetic study also revealed the relationship between *E. variegata* and related species in Fabaceae.

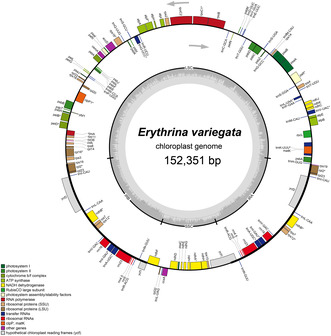

## 1 Introduction

Chloroplast is an essential organelle of angiosperms that is responsible for photosynthesis (Shanker et al. 2022). Chloroplast has its own genomes (cpDNA) that are usually 120–160 kb in length and encode 80 protein‐coding genes, 30 transfer RNA genes, and four ribosomal RNA genes (Daniell et al. 2016). The genomic information of the chloroplast is crucial for elucidating the evolutionary history of angiosperms. For example, the backbone phylogenetic tree of land plants and related species was established based on the plastome data (Gitzendanner et al. 2018). Additionally, the sequences of cpDNA were used for mining molecular markers, exploring population genetics, and developing biotechnology (Kumar and Ling 2021; Li, Zheng, and Huang 2020; Olejniczak et al. 2016; Song et al. 2023). It is clear that chloroplast genome data are precious for genomic studies of angiosperms.

*Erythrina* L. is a genus of Fabaceae and contains 124 species distributed in tropical and subtropical regions (POWO 2024). Members of *Erythrina* possess various metabolites for flavonoids, alkaloids, and triterpenoids; therefore, they exhibit anti‐inflammatory, antidiabetic, estrogenic, antifungal, antidepressant, anticancer, and antibacterial activities (Susilawati et al. 2023). For example, the lectin extracted from seeds of *Erythrina senegalensis* exhibited antimicrobial activities (Enoma et al. 2023). Similarly, the bark of *Erythrina suberosa* had cytotoxic and antimicrobial features (Ahmed et al. 2020). A similar pattern was also found in *E. variegata* of which the bark contained alkaloids, phenols, and flavonoids and showed an enhancement of memory, anti‐inflammatory, and antidiabetic activities (Biradar et al. 2022; Santhiya et al. 2016). In various African countries, *Erythrina abyssinica* was used as a traditional herbal medicine for treatment of malaria, bacterial infection, skin injury, diarrhea, tuberculosis, diabetes, and meningitis (Obakiro et al. 2021). Another example is the traditional use of *Erythrina velutina* in Brazil for insomnia, toothache, headache, cough, and fever (Adetunji et al. 2024). Last but not least, traditional medicines made of *Erythrina variegata* plant were used for anti‐asthmatic, antiepileptic, antiseptic, and toothache treatments in India (Karunanithi, Rajkishore, and Radharamalingam 2017). Despite reported pharmacological features, only three chloroplast genomes of *Erythrina* species have been reported (Choi et al. 2022; McAssey et al. 2023; Oyebanji et al. 2020). The current genomic data of *Erythrina* genus is deficient to elucidate its evolutionary history. Therefore, in this study, we sequenced and characterized the complete chloroplast genome of *Erythrina variegata* to enlarge the genomic data of *Erythrina* genus and to provide essential information to trace the evolutionary pattern of this interesting genus in further studies. Additionally, phylogenetic relationships between *E. variegata* and related species in Fabaceae were reconstructed.

## 2 Materials and Methods

**FIGURE 1:**
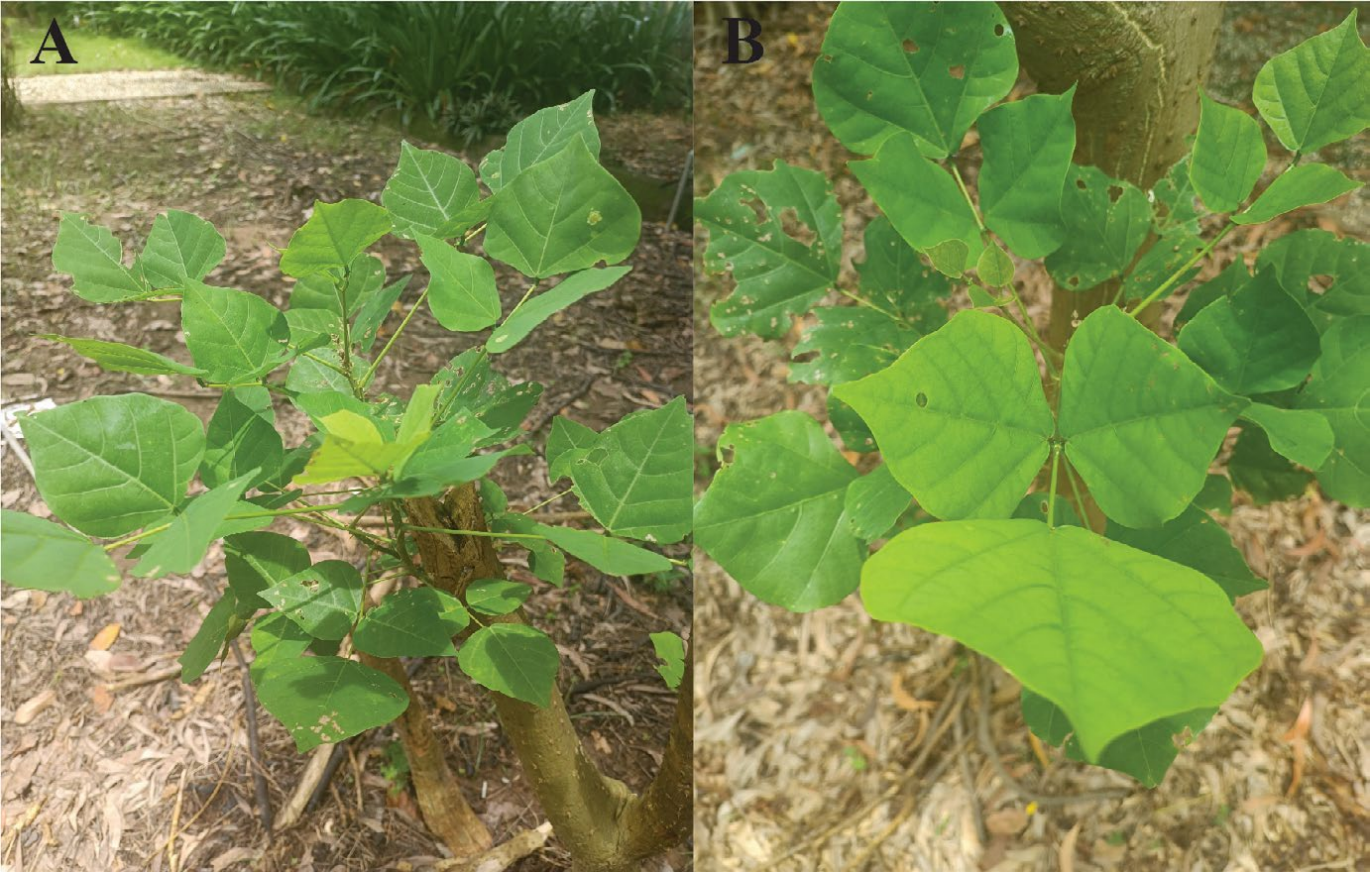
Illustration of *Erythrina variegata* in the field. (A) The whole plant. (B) The leaves. The tree (with a diameter of approximately 10 cm) was cut down but the young branches emerged. The trunk has small thorns. The trifoliate leaves are alternate and have two leaflets that are arranged opposite, of which the terminal leaf (ovate to rhomboid) is larger than the lateral ones. Photo taken by Nguyen Nhat Nam at Tra Vinh University, Tra Vinh, Viet Nam.

The fresh leaves of *Erythrina vagiegata* were collected at Tra Vinh University (9°55′25.1″ N/106°20′47.5″ E) and dried with silica gel beads (Figure 1). The dried leaves were stored at −81°C for further experiments. The sample was deposited to School of Agriculture and Aquaculture, Tra Vinh University under voucher number of SAA‐TVU‐2024‐0110147 (contact person: Dr. Nguyen Nhat Nam, nnnam@tvu.edu.vn). The total genomic DNA was extracted using DNeasy Plant Pro Kits (Qiagen, USA) following the manufacturer's instructions. The quality of DNA samples was checked using NanoDrop One Microvolume UV–Vis Spectrophotometer (Thermo Fisher Scientific, USA) and gel electrophoresis. The high‐quality DNA sample (having a concentration of at least 100 ng/μL and a clear band on the 1% agarose gel) was applied for the Nextseq 550 sequencing system (Illumina, USA) to generate 150 bp paired‐end reads.

The raw sequencing data were checked and filtered using fastp v0.23.4 with default options for trimming and filtering processes (Chen et al. 2018). Then, NOVOPlasty v4.5.3 was used to assemble the complete chloroplast genome of *E. variegata* with the chloroplast genome of *E. herbacea* (GenBank accession number OL672880) as a reference and default settings (Dierckxsens, Mardulyn, and Smits 2016). The gene content of the newly completed cpDNA was annotated using Geseq (Tillich et al. 2017) and then checked using Geneious Prime v2024.2 (www.geneious.com) and tRNAscan‐SE 2.0 (Chan et al. 2021). The map of the cpDNA and illustration of trans‐ and cis‐splicing genes were generated using OGDRAW v1.3.1 and CPGView, respectively (Greiner, Lehwark, and Bock 2019; Liu et al. 2023).

For phylogenetic analysis, the complete chloroplast genomes of 13 species were downloaded from the GenBank database with *Robinia pseudoacacia* (GenBank accession number KJ468102) serving as an outgroup according to a previous phylogenetic study of papilionoid legume (Table 1) (Choi et al. 2022). A total of 78 protein‐coding regions (except *infA* gene which is pseudogenized and lost in the surveyed taxa) were extracted and aligned using Muscle5 embedded in Geneious Prime v2024.2 (Edgar 2022). The aligned sequences were subjected to jModeltest2 v2.1.10 to identify the best substitution model for elucidating the phylogeny with default settings and Best option for Base tree search (Darriba et al. 2012). Consequently, the model of GTR + I + G (Akaike Information Criterion) was chosen for running phylogenetic analysis using maximum likelihood (ML) and Bayesian inference (BI) methods. The ML method was conducted using IQ‐TREE 2 with substitution model of GTR + I + G, ultrafast option for bootstrap analysis with 1000 replicates and other default settings (Minh et al. 2020). Meanwhile, the BI method was performed using MrBayes v3.2.7 with GTR + I + G substitution model and 1,000,000 generations (Ronquist et al. 2012). Additionally, 25% of samples generated by MrBayes were discarded by default. The phylogenetic trees were illustrated using Figtree v1.4.4 (http://tree.bio.ed.ac.uk/software/figtree/).

**TABLE 1:** List of species used for phylogenetic analysis.

Species	Accession number	Tribe	Subfamily	Family
*Robinia pseudoacacia*	KJ468102	Robinieae	Papilionoideae	Fabaceae
*Indigofera tinctoria*	KJ468098	Indigofereae	Papilionoideae	Fabaceae
*Platycyamus regnellii*	OL672877	Millettieae	Papilionoideae	Fabaceae
*Cullen corylifolium*	MK069582	Psoraleeae	Papilionoideae	Fabaceae
*Campylotropis macrocarpa*	MG867566	Desmodieae	Papilionoideae	Fabaceae
*Phyllodium pulchellum*	MN614126	Desmodieae	Papilionoideae	Fabaceae
*Hylodesmum podocarpum*	MT120798	Desmodieae	Papilionoideae	Fabaceae
*Psophocarpus tetragonolobus*	MN966643	Phaseoleae	Papilionoideae	Fabaceae
*Phaseolus acutifolius*	OL672884	Phaseoleae	Papilionoideae	Fabaceae
*Apios americana*	KF856618	Phaseoleae	Papilionoideae	Fabaceae
*Erythrina sandwicensis*	OQ870895	Phaseoleae	Papilionoideae	Fabaceae
*Erythrina herbacea*	OL672880	Phaseoleae	Papilionoideae	Fabaceae
*Erythrina crista‐galli*	MN966629	Phaseoleae	Papilionoideae	Fabaceae
*Erythrina variegata*	PQ469842	Phaseoleae	Papilionoideae	Fabaceae

## 3 Results and Discussion

**FIGURE 2:**
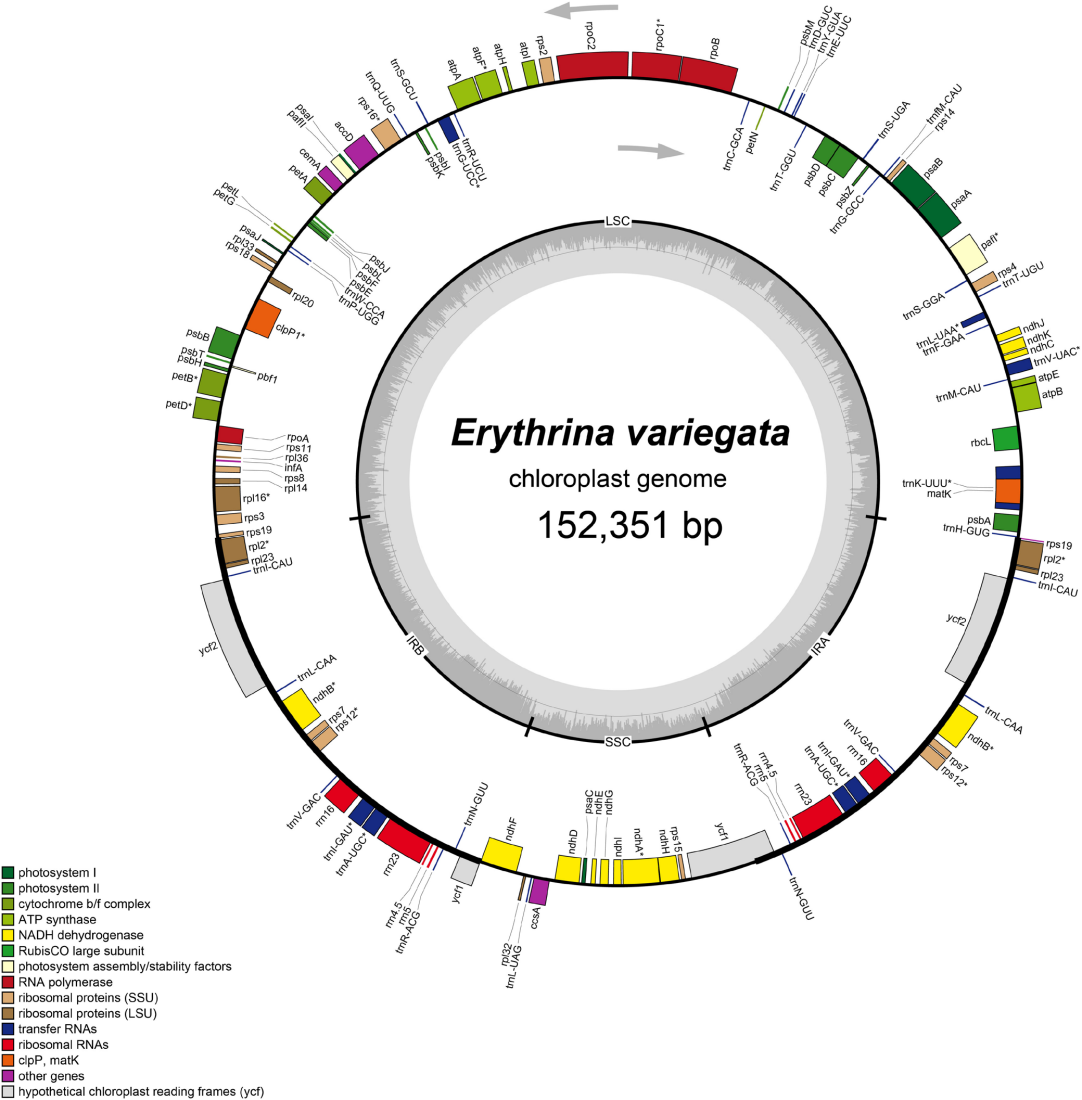
Map of the chloroplast genome of *Erythrina variegata* . The arrows indicated the translation directions of inner and outer genes. The inner circle indicates the GC contents. The asterisks mean the gene having intron. IRA and IRB, Inverted repeat regions; LSC, Large single copy; SSC, Small single copy.

**FIGURE 3:**
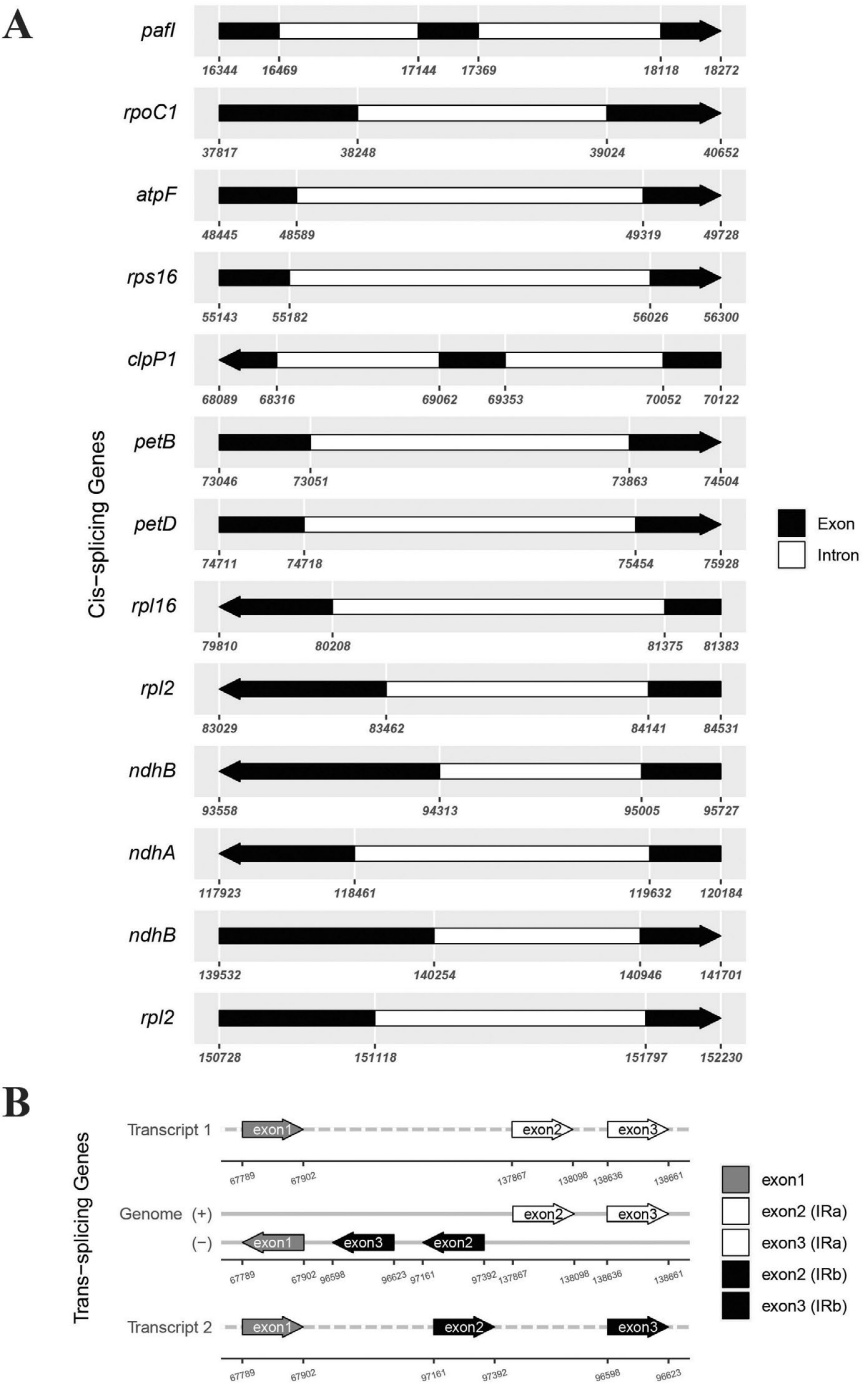
Schematic map of the cis and trans‐splicing genes in the chloroplast genomes of *Erythrina variegata* . (A) The cis‐splicing genes. (B) The trans‐splicing genes.

The complete chloroplast genome of *E. variegata* (average coverage 618×) was 152,351 bp in length and had a quadripartite structure (Figure 2). This genome consisted of four regions, including a large single copy (LSC), a small single copy (SSC), and two inverted repeat (IR) regions, of which the lengths were 82,907 bp, 16,826 bp, and 26,309 bp, respectively. Additionally, the cpDNA of *E. variegata* was composed of 79 protein‐coding genes, 30 transfer RNA genes, and four ribosomal RNA genes (Table 2). There were 19 duplicated genes in the IR regions, including *rrn4.5*, *rrn5*, *rrn16*, *rrn23*, *trnA‐UGC*, *trnL‐CAA*, *trnI‐GAU*, *trnI‐CAU*, *trnN‐GUU*, *trnR‐ACG*, *trnV‐GAC*, *rpl2*, *rpl22*, *rps7*, *rps12*, *rps19*, *ndhB*, *ycf1*, and *ycf2*. Additionally, *trnA‐UGC*, *trnG‐UCC*, *trnI‐GAU*, *trnK‐UUU*, *trnL‐UAA*, *trnV‐UAC*, *rpl2*, *rpl16*, *rps16*, *rpoC1*, *petB*, *petD*, *atpF*, *ndhA*, and *ndhB* had one intron whereas *pafI* and *clpP1* had two introns (Figure 3A). The *rps12* gene was a trans‐splicing gene, of which exon 2 and exon 3 were located in the IR regions (Figure 3B).

**TABLE 2:** Gene composition of *Erythrina variegata* chloroplast genome.

Groups of genes	Name of genes
Ribosomal RNAs	*rrn4.5* ^*a*^, *rrn5* ^*a*^, *rrn16* ^*a*^, *rrn23* ^*a*^
Transfer RNAs	*trnA‐UGC* ^a^, ^b^, *trnC‐GCA, trnD‐GUC, trnE‐UUC, trnF‐GAA, trnG‐UCC* ^*b*^, *trnG‐GCC, trnH‐GUG, trnI‐GAU* ^a^, ^b^, *trnK‐UUU* ^*b*^, *trnL‐CAA* ^*a*^, *trnL‐UAA* ^*b*^, *trnL‐UAG, trnfM‐CAU, trnI‐CAU* ^*a*^, *trnM‐CAU, trnN‐GUU* ^*a*^, *trnP‐UGG, trnQ‐UUG, trnR‐ACG* ^*a*^, *trnR‐UCU, trnS‐GCU, trnS‐GGA, trnS‐UGA, trnT‐GGU, trnT‐UGU, trnV‐GAC* ^*a*^, *trnV‐UAC* ^*b*^, *trnW‐CCA, trnY‐GUA*
Large units of ribosome	*rpl2* ^a^, ^b^, *rpl14, rpl16* ^*b*^, *rpl20, rpl22* ^*a*^, *rpl23, rpl32, rpl33, rpl36*
Small units of ribosome	*rps2, rps3, rps4, rps7* ^*a*^, *rps8, rps11, rps12* ^a^, ^c^, *rps14, rps15, rps16* ^*b*^, *rps18, rps19* ^*a*^
RNA polymerase	*rpoA, rpoB, rpoC1* ^*b*^, *rpoC2*
Translational initiation factor	*infA*
Subunit of photosystem I	*psaA, psaB, psaC, psaI, psaJ, pafI* ^*c*^, *pafII*
Subunit of photosystem II	*psbA, psbB, psbC, psbD, psbE, psbF, psbH, psbI, psbJ, psbK, psbL, pbfI, psbM, psbT, psbZ*
Subunit of cytochrome	*petA, petB* ^*b*^, *petD* ^*b*^, *petG, petL, petN*
Subunit of ATP synthases	*atpA, atpB, atpE, atpF* ^*b*^, *atpH, atpI*
Large unit of Rubisco	*rbcL*
Subunit of NADH dehydrogenase	*ndhA* ^*b*^, *ndhB* ^a^, ^b^, *ndhC, ndhD, ndhE, ndhF, ndhG, ndhH, ndhI, ndhJ, ndhK*
Maturase	*matK*
Envelope membrane protein	*cemA*
Subunit of acetyl‐CoA	*accD*
C‐type cytochrome synthesis gene	*ccsA*
ATP‐dependent protease subunit P	*clpP1* ^*c*^
Hypothetical proteins and conserved reading frames	*ycf1* ^*a*^, *ycf2* ^*a*^

^a^ Duplicated gene in IR region.

^b^ Genes containing single intron.

^c^ Genes containing two introns.

Comparative analysis revealed high conservation in chloroplast genomes of examined *Erythrina* species regarding gene content and order, genome structure, GC content, and genome size (Table 3). Although an identical number of genes was found, the cpDNAs of four *Erythrina* taxa were slightly different in length. Specifically, the biggest cpDNA belonged to *E. variegata* whereas *E. crista‐galli* had the smallest cpDNA. Additionally, the size of LSC, SSC, and IR regions ranged from 82,389 bp to 82,907 bp, from 16,756 bp to 16,826 bp, and from 26,303 bp to 26,360 bp respectively. Among four *Erythrina* cpDNA, the junction between LSC and IR regions was mostly located in *rps19* genes. Only *E. sandwicensis* had the LSC/IR junction in the intergenic spacer between *rps19* and *rpl22* genes. In the case of the boundary between the SSC and IR regions, three species exhibited the overlap of *ycf1* and *ndhF* genes. In contrast, the SSC/IR junction of *E. crista‐galli* was located within *ycf1* gene (Table 3).

**TABLE 3:** Features of chloroplast genomes among *Erythrina* species.

Species	*Erythrina sandwicensis*	*Erythrina herbacea*	*Erythrina crista‐galli*	*Erythrina variegata*
Accession number	OQ870895	OL672880	MN966629	PQ469842
Total length (bp)	152,399	151,821	151,751	152,351
Total % GC	35.1	35.3	35.3	35.1
LSC length (bp)	82,865	82,403	82,389	82,907
LSC % GC	32.4	32.7	32.8	32.5
SSC length (bp)	16,814	16,784	16,756	16,826
SSC % GC	28.3	28.7	28.8	28.4
IR length (bp)	26,360	26,317	26,303	26,309
IR % GC	41.3	41.4	41.4	41.4
Protein‐coding gene	79	79	79	79
tRNAs	30	30	30	30
rRNAs	4	4	4	4
LSC/IR junction	IGS (*rps19‐rpl22*)	*rps19* (68 bp)	*rps19* (68 bp)	*rps19* (67 bp)
SSC/IR junction	Overlap (*ycf1*–1295 bp/*ndhF*—28 bp)	Overlap (*ycf1*–1280 bp/*ndhF*—2 bp)	*ycf1* (1249 bp)	Overlap (*ycf1*–1280 bp/*ndhF*—2 bp)

Previous studies examining chloroplast genomes in Fabaceae demonstrated the conservation of genomic features within the genera. For example, comparative chloroplast genome analyses of six *Hedysarum* and 22 *Campylotropis* confirmed the quadripartite structure or loss of one IR region, gene content, and genome size within these genera (Feng et al. 2022; Juramurodov et al. 2023). Similarly, another comparative genomic analysis of 35 *Dalbergia* species disclosed the similarity of genome features, including junctions between LSC, SSC, and IR regions, the numbers of protein‐coding genes, tRNA genes, and rRNA genes, and codon usage (Hong et al. 2022). These previous results demonstrated the high conservation of chloroplast genomes among the genera of Fabaceae. In the current study, the phenomenon of conservation was also found; however, only four *Erythrina* species were observed. Therefore, further studies examining 124 members of *Erythrina* species should be conducted to explore the evolutionary history of this interesting genus of Fabaceae.

**FIGURE 4:**
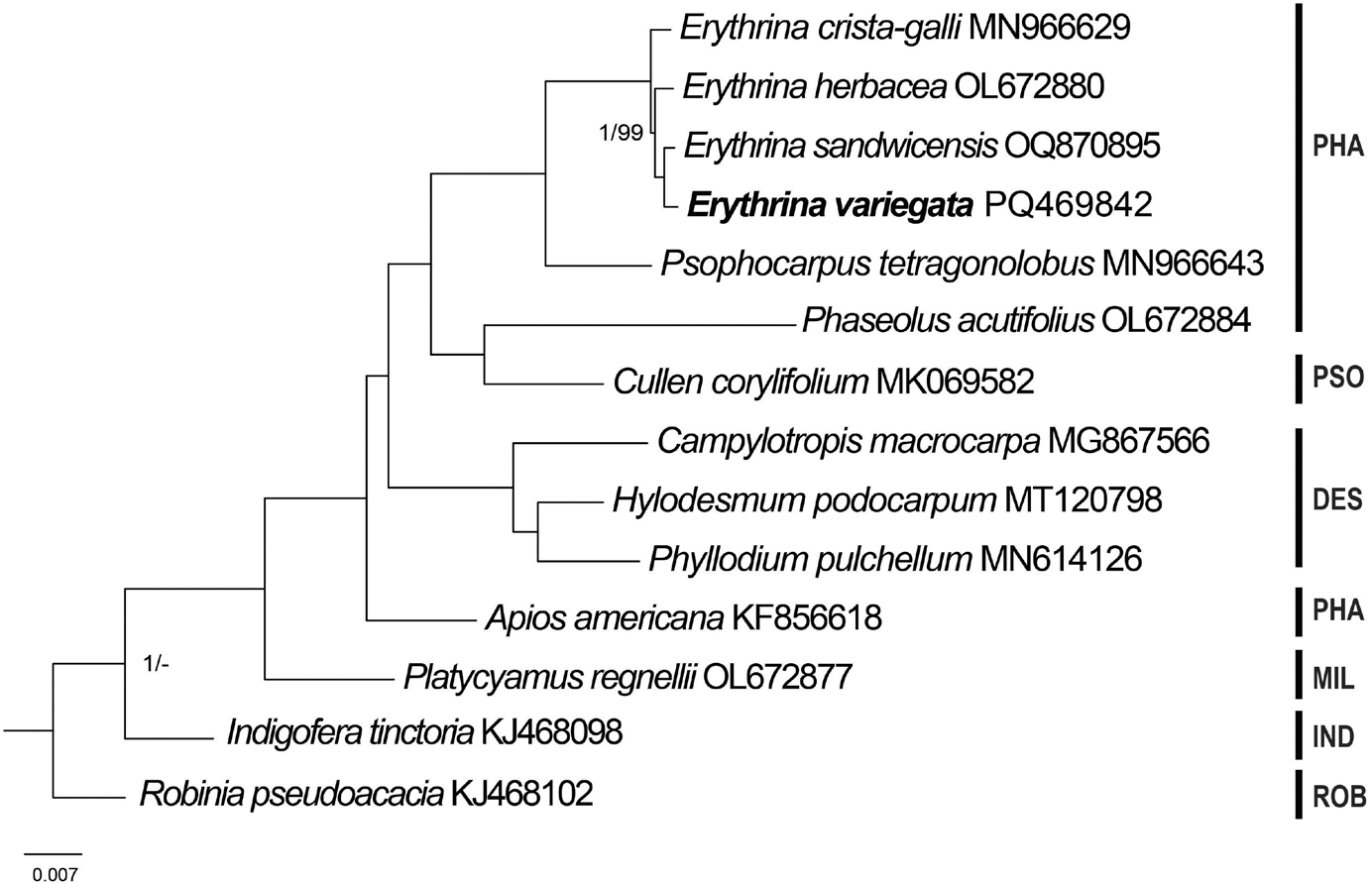
Phylogenetic relationship of *Erythrina variegata* and related species inferred from 78 protein‐coding genes using maximum likelihood and Bayesian Inference methods. Only bootstrap values smaller than 100 and posterior probability under 1 were shown at the nodes. DES, Desmodieae; IND, Indigofereae; MIL, Millettieae; PHA, Phaseoleae; PSO, Psoraleeae; ROB, Robinieae.

The ML and BI methods resulted in the same topology of phylogenetic trees (Figure 4). The monophyly of *Erythrina* species was highly supported (bootstrap value = 100 and posterior probability = 1). Within the *Erythrina* genus, *E. variegata* had a close relationship with *E. sanwicensis*. However, members of the Phaseoleae tribe exhibited a polyphyletic trend, in which *Phaseolus acutifolius* formed a clade with *Cullen corylifolium* of the Psoraleeae tribe. Similarly, *Apios americana* was sister to groups of Desmodieae, Phaseoleae, and Psoraleeae tribes. The non‐monophyletic patterns of members in Papilionoideae were also found in previous studies based on genomic and transcriptomic data (Choi et al. 2022; Zhao et al. 2021). These results raised a question about the tribal classification of Papilionoideae and require more data in further studies to fulfill the taxonomic treatments within Fabaceae.

## 4 Conclusion

In the current study, we reported the complete chloroplast genome of *Erythrina variegata* using the next‐generation sequencing method. Comparative genomic analysis revealed the similarities and differences among four *Erythrina* species regarding genome size, genome structure, GC content, and gene content. Additional phylogenetic analysis provided an initial relationship between *E. variegata* and related taxa based on 78 protein‐coding regions of cpDNA. The outcomes of this study provided essential information for further genomic studies of *E. variegata* and other members of Fabaceae.

## Author Contributions

**Nguyen Nhat Nam:** conceptualization (equal), data curation (lead), formal analysis (equal), writing – original draft (lead). **Nguyen Ngoc Trai:** data curation (supporting), methodology (supporting), software (supporting), visualization (equal), writing – review and editing (supporting). **Nguyen Phuong Thuy:** conceptualization (equal), methodology (supporting), software (supporting), visualization (equal). **Le Quoc Duy:** methodology (supporting), visualization (equal), writing – review and editing (supporting). **Pham Nguyen Tuong Van:** methodology (supporting), visualization (equal), writing – review and editing (supporting). **Tan Tai Nguyen:** conceptualization (equal), investigation (equal), writing – review and editing (supporting). **Hoang Dang Khoa Do:** conceptualization (equal), formal analysis (equal), methodology (lead), software (lead), writing – review and editing (supporting).

## Conflicts of Interest

The authors declare no conflicts of interest. No permission is needed to collect the samples of *E. variegata* for research purposes at Tra Vinh University.

## Data Availability

The complete chloroplast genome of *Erythrina variegata* was submitted to GenBank under accession number PQ469842. The raw sequencing data were also deposited to NCBI under the accession number PRJNA1183432.
